# Changes in Lumbo-Pelvic Coordination of Individuals With and Without Low Back Pain When Wearing a Hip Orthosis

**DOI:** 10.3389/fspor.2020.00090

**Published:** 2020-07-16

**Authors:** Matthew T. Ballard, Colin Drury, Babak Bazrgari

**Affiliations:** F. Joseph Halcomb III, M.D. Department of Biomedical Engineering, University of Kentucky, Lexington, KY, United States

**Keywords:** low back pain, lumbo-pelvic coordination, orthosis, lumbopelvic rhythm, lumbar rotation, pelvic rotation

## Abstract

Individuals with low back pain demonstrate an abnormal lumbo-pelvic coordination compared to back-healthy individuals. This abnormal coordination presents itself as a reduction in lumbar contributions and an increase in pelvic rotations during a trunk forward bending and backward return task. This study investigated the ability of a hip orthosis in correcting such an abnormal lumbo-pelvic coordination by restricting pelvic rotation and, hence increasing lumbar contributions. The effects of the hip orthosis on the lumbo-pelvic coordination were investigated in 20 low back pain patients and 20 asymptomatic controls. The orthosis reduced pelvic rotation by 12.7° and increased lumbar contributions by 11%. Contrary to our expectation, orthosis-induced changes in lumbo-pelvic coordination were smaller in patients; most likely because our relatively young patient group had smaller unrestricted pelvic rotations compared to asymptomatic individuals. Considering the observed capability of a hip orthosis in causing the expected changes in lumbo-pelvic coordination when there is a relatively large pelvic contribution to trunk motion, application of a hip orthosis may provide a promising method of correcting abnormal lumbo-pelvic coordination, particularly among patients who demonstrate larger pelvic rotation, that warrants further investigation.

## Introduction

Over one-third of individuals are afflicted with low back pain (LBP) every year (Hoy et al., 2012), and recurrence rates have been cited as high as 44% within 1 year of onset (Woolf and Pfleger, 2003). A major challenge in treatment of a patient suffering from LBP is the difficulty to determine a root cause using current diagnostic approaches. Most cases are categorized as non-specific LBP, with specific diagnoses only assigned in an estimated 10% of cases (Krismer and Van Tulder, 2007). Such a challenge has motivated much research on differences in factors known to have a role in an individual's experience of LBP between individuals with and without LBP. For instance, lower back biomechanics has been suggested to have a causal role in LBP (Adams et al., 2006). Subsequently, many researchers investigated differences in factors contributing to lower back biomechanics between individuals with and without LBP. This included studies on differences in activities of trunk muscles (Ahern et al., 1988; Paquet et al., 1994), trunk motion and lumbo-pelvic coordination (LPC) (Mayer et al., 1984; Marras and Wongsam, 1986; Porter and Wilkinson, 1997; Thomas and France, 2008; Thomas et al., 2008), and spinal loads (Kumar, 1990; Neumann et al., 2001). The outcomes of such studies (i.e., the identified abnormalities in different aspects of lower back biomechanics of patients with LBP) then motivated other studies on treatments aimed at modification of such specific abnormalities in lower back biomechanics (Hoffman et al., 2011; Morrisette et al., 2014; Smith et al., 2014; Searle et al., 2015; Shahvarpour et al., 2017).

LPC is defined as the relative contribution of lumbar and pelvic rotations to total trunk motion and is often examined qualitatively in a clinical setting during a forward bending and backward return task. Quantitatively in research labs, LPC has been characterized using several different approaches and by determining the timing, coordination and magnitude of lumbar and pelvic contributions to total trunk motion throughout a task including but not limited to forward bending and backward return task (Vazirian et al., 2016a). Emerging from these earlier investigations and despite some discrepancies, LBP patients appear to demonstrate an altered LPC involving smaller lumbar and larger pelvic contributions to trunk motion when compared to asymptomatic individuals (Marras and Wongsam, 1986; Ahern et al., 1988; Paquet et al., 1994; Porter and Wilkinson, 1997; Shojaei et al., 2017a). Additionally, the timing and coordination of pelvic and lumbar movements is altered among patients with LBP (Vazirian et al., 2016b). Furthermore, such an abnormal LPC of patients with LBP has been suggested to persist even after symptoms have subsided (Ferguson et al., 2000; Thomas and France, 2008; Shojaei et al., 2019). Retaining such abnormalities in LPC may prove problematic due to resultant increases in spinal loading. Biomechanical modeling has shown a decrease in lumbar contributions to trunk forward bending and backward return would increase compression and shear forces in the spine (Tafazzol et al., 2014). Additionally, such an abnormal LPC has been found to cause higher shearing demands in the lower back while handling a small load (4.5 kg) during trunk flexion (Shojaei et al., 2018). Although an abnormal LPC at presence of LBP might in part be considered a result of pain, its persistence beyond symptom recovery, particularly considering its negative impact on lower back biomechanics and spinal loads, is likely to be associated with higher risk of LBP recurrence. Given the high recurrence rate of LBP, it is worth investigating whether correction of abnormal LPC of patient with LBP could play a role in reducing subsequent LBP occurrences.

Back orthoses (a.k.a. back belts) are commonly used by individuals with a recent back injury, and have been shown to correlate with short-term reduction in LBP symptoms (Larivière et al., 2014); however, the purpose of these devices is to reduce lumbar range of motion in attempt to prevent injury from overuse. Therefore, back orthoses are not suitable for correction of the above noted abnormalities in LPC of patients with LBP that have been widely reported to be associated with a reduced lumbar motion. Similarly, exercise programs commonly employed in physical therapy have shown correlation with a reduction in LBP symptoms in chronic patients (Searle et al., 2015), but they often are ineffective at correcting abnormal LPC (Shahvarpour et al., 2017). Other physical therapy techniques have shown some effectiveness in LPC correction, with patients who are able to achieve the correction reporting lower pain ratings than those unable to correct (Mayer et al., 2009). This supports the pursuit of LPC correction, but as current methods have not been shown effective against all patients, new methods are worth examining.

Given that LBP patients with abnormal LPC have smaller lumbar contributions and larger pelvic contributions, it may be possible to increase lumbar contributions (and correct LPC) by restricting pelvic movement. If these individuals are relying on increases in pelvic movements to complete tasks, adding restriction to the pelvis may encourage them to use more lumbar motion. The objective of this study was to determine whether using a hip orthosis to restrict pelvic rotation would drive more lumbar contributions to trunk motion during a trunk forward bending task. The hypotheses tested are (1) the orthosis will change LPC by limiting pelvic rotation hence leading to larger lumbar contribution to trunk motion; and (2) the orthosis-induced changes in LPC will be larger in patients with LBP compared to back healthy controls. As an exploratory aspect of the study, the effects of orthosis on timing and coordination aspects of LPC were also investigated. If successful, application of hip orthosis could be a useful approach in training LBP patients to regain an LPC similar to back healthy individuals.

## Methods

### Study Design and Participants

A repeated measures design was used to evaluate the effects of pelvic motion restriction, using a hip orthosis, on LPC during a trunk forward bending and backward return task. Twenty individuals with a recent history or a current episode of LBP (LBP; 10M, 10F) and twenty asymptomatic controls (healthy; 11M, 9F) were recruited (Table 1). In an effort to eliminate factors other than LBP and the hip orthosis which may affect LPC, prospective individuals were excluded for presence of musculoskeletal or neuromuscular disorders (other than LBP), current musculoskeletal injuries, or history of spinal surgery. Before participation in the study, each individual underwent an informed consent and screening process that was approved by the University of Kentucky Institutional Review Board.

**Table 1:** Mean *(SD)* of mass (kg), stature (cm), and age (year) for participants.

	**Subject Demographics** ***(SD)***		
	**Healthy**	**LBP**	***P*-value**
Weight (kg)	78.04 (17.51)	81.86 (19.95)	0.524
Stature (cm)	172.33 (7.74)	171.33 (8.6)	0.701
Age	22.7 (3.37)	21.05 (2.89)	0.105
Pain	N/A	4.4 (1.3)	

*Anthropometric data for each group compared using an independent samples t-test; p < 0.05 indicates significance. A numerical rating scale (0–10) was used to record the level of pain of participants with LBP during the data collection (Hjermstad et al., 2011)*.

### Experimental Procedures

At the start of the experiment, participants were instrumented with wireless, tri-axial inertial measurement units (IMUs; Xsens Technologies, Enschede, Netherlands) placed superficially over the T10 (thorax) and the S1 (pelvis) vertebrae using Velcro straps (Shojaei et al., 2017a,b, 2019; Vazirian et al., 2017a). The IMUs were assumed to measure the rotations of the thorax and pelvis as rigid bodies. Once the IMUs were placed on the participant, their positions were maintained throughout the entire experiments without disturbance to maintain accuracy across conditions.

Each participant then completed the forward bending and backward return task under two conditions in a random order: with and without the orthosis. Participants were instructed to cross their arms over their chest, keep their knees straight and feet stationary throughout the procedure. An audible cue would be given for the individual to bend forward and hold at maximum range of motion until a second cue was given to return to the upright position. The time spent at maximum range of motion and the time between repetitions of the task was ~5 s. The task was completed a minimum of six times, after which the orthosis would be placed on, or removed from, the participant for the alternate condition. Prior to data collection, research personnel demonstrated the task and participants were given the opportunity to practice.

**Figure 1:**
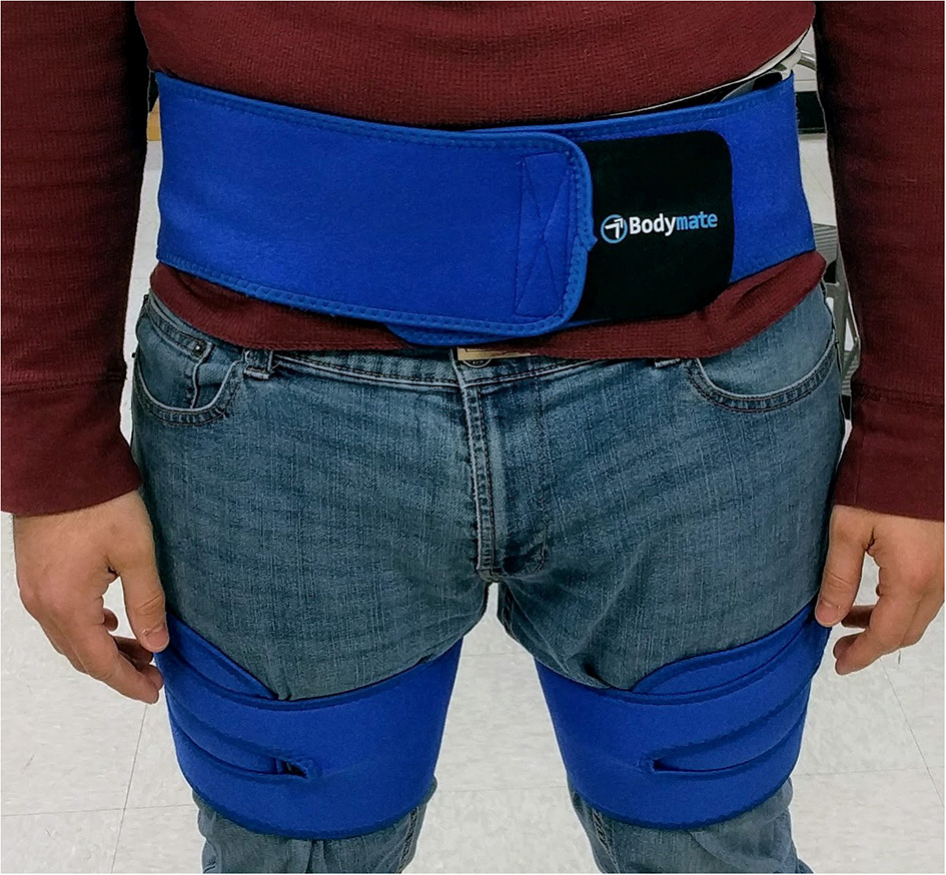
A Hip Orthosis (BodyMate, CA, USA) fabricated from flexible, neoprene material was used to constrain the pelvic rotation.

The hip orthosis used was a compression wrap constructed of flexible, neoprene material (BodyMate, CA, USA) that attaches via hook and loop fasteners around the waist and thighs (Figure 1). The same orthosis was used for all participants.

### Data Collection and Analysis

Three-dimensional orientation data are collected from the IMUs using MT Manager software (Xsens Technologies, Enschede, Netherlands). Data are sampled at a rate of 60 Hz and passed through a Kalman filter to minimize noise. A custom script in MATLAB (MathWorks, MA, USA) was employed to extract the rotations of the thorax and pelvis in the sagittal plane from the IMU data.

Details of methods used to calculate thoracic and pelvic rotation can be found in Vazirian et al. (2019), but briefly, the rotation matrices extracted from the IMUs were used to obtain rotation quaternions (a rotation about a unit vector n through an angle α for each IMU) and then to calculate the pelvic and thoracic rotations in the sagittal plane. The initial standing posture was regarded as the reference posture. At each time point, lumbar flexion was calculated from the difference between the thoracic and pelvic rotations.

#### Magnitude Aspect

The values of the following measures at the time of maximum thoracic rotation were extracted for evaluating the magnitude aspect of LPC: (1) thoracic rotation; (2) pelvic rotation; (3) lumbar rotation; and (4) lumbar-thoracic ratio (LTR). LTR was calculated by dividing the lumbar rotation by thoracic rotation. As thoracic rotation represents the overall trunk range of motion, LTR (presented as a percentage) represents the lumbar contribution to the overall trunk motion. LTR serves as a measure that is independent of individual variations in total range of motion. For each measure, its value at the time of the maximum thoracic rotation was first obtained for each cycle of the task and then averaged across all repetitions of the task. In this process, all the measures associated with a given cycle were excluded from averaging (i.e., marked as outlier) if a measure in that cycle was found to have a value that was off more than three standard deviations from the respective mean value of that measure.

#### Timing Aspect

Mean absolute relative phase (MARP) and deviation phase (DP) were values used to represent the timing aspect of LPC. MARP and DP were extracted from a continuous relative phase signal as in Vazirian et al. (2017b). Rotation matrices obtained from the thorax and pelvis IMUs were used to generate thorax and pelvis phase planes according to Lamb and Stöckl (2014). For each task (bending and return) a reference point was first selected between the standing and maximum range-of-motion position such that the two end positions would have equally negative and positive values. The rotation signals were transformed using the Hilbert transform. The rotation signals were then plotted against their Hilbert transforms to generate the thorax and pelvic phase planes. At each time point, the continuous relative phase was calculated by subtracting the pelvic phase angle from the thoracic phase angle. For each participant, the continuous relative phase for all repetitions of the task are used to calculate a mean and standard deviation for each percentile of the task. The MARP and DP are the average of the calculated mean and standard deviation, respectively.

#### Coordination Pattern

It has been suggested that information obtained from continuous relative phase (e.g., MARP and DP) are limited to phase relationship between two segments and do not offer insight related to the dominancy of one segment's motion over the other one's (Needham et al., 2014). Therefore, a vector coding technique previously described (Needham et al., 2014) was used to analyze the coordination between pelvis and lumbar flexion during the task. In this approach, the lumbar and pelvic rotation data were first separated into two phases (bending and return) for each repetition and normalized to 100 points for each percentile of motion. For each phase, a plot of pelvic rotation vs. lumbar rotation was generated. Each plot was then used to calculate coupling angles, defined as the angle of a vector from each time point to the next, relative to the right horizontal. For each phase, coupling angles were averaged across all points for all repetitions to obtain one value of coupling angle. This was repeated for all subjects under each condition. Coupling angle variability (CAV) was also found for each point using rotational statistics and averaged to find one value for each phase. The value of the coupling angle allows the classification of LPC (Needham et al., 2015) into one of four categories: (1) in-phase with proximal (lumbar) dominancy; (2) in-phase with distal (pelvic) dominancy; (3) anti-phase with proximal dominancy; or (4) anti-phase with distal dominancy. In-phase refers to the two segments moving in the same direction, while anti-phase refers to them moving in opposite directions.

### Statistical Methods

Dependent variables for the magnitude aspect (thoracic rotation, pelvic rotation, lumbar rotation, LTR), timing aspect (bending MARP, return MARP, bending DP, return DP), and the coordination pattern (bending coupling angle, return coupling angle, bending CAV, return CAV) were analyzed using a repeated measure, mixed factor analysis of variance (ANOVA) test. In the comparisons, orthosis condition (with or without orthosis) served as the within-subjects factor while group (healthy or LBP) served as the between-subjects factor. A *p* < 0.05 was used to determine significance. *Post-hoc* analyses were performed using paired and un-paired sample *t*-tests (for condition and group effects, respectively) with an adjusted *p* < 0.0125 to determine significance.

## Results

**Figure 2:**
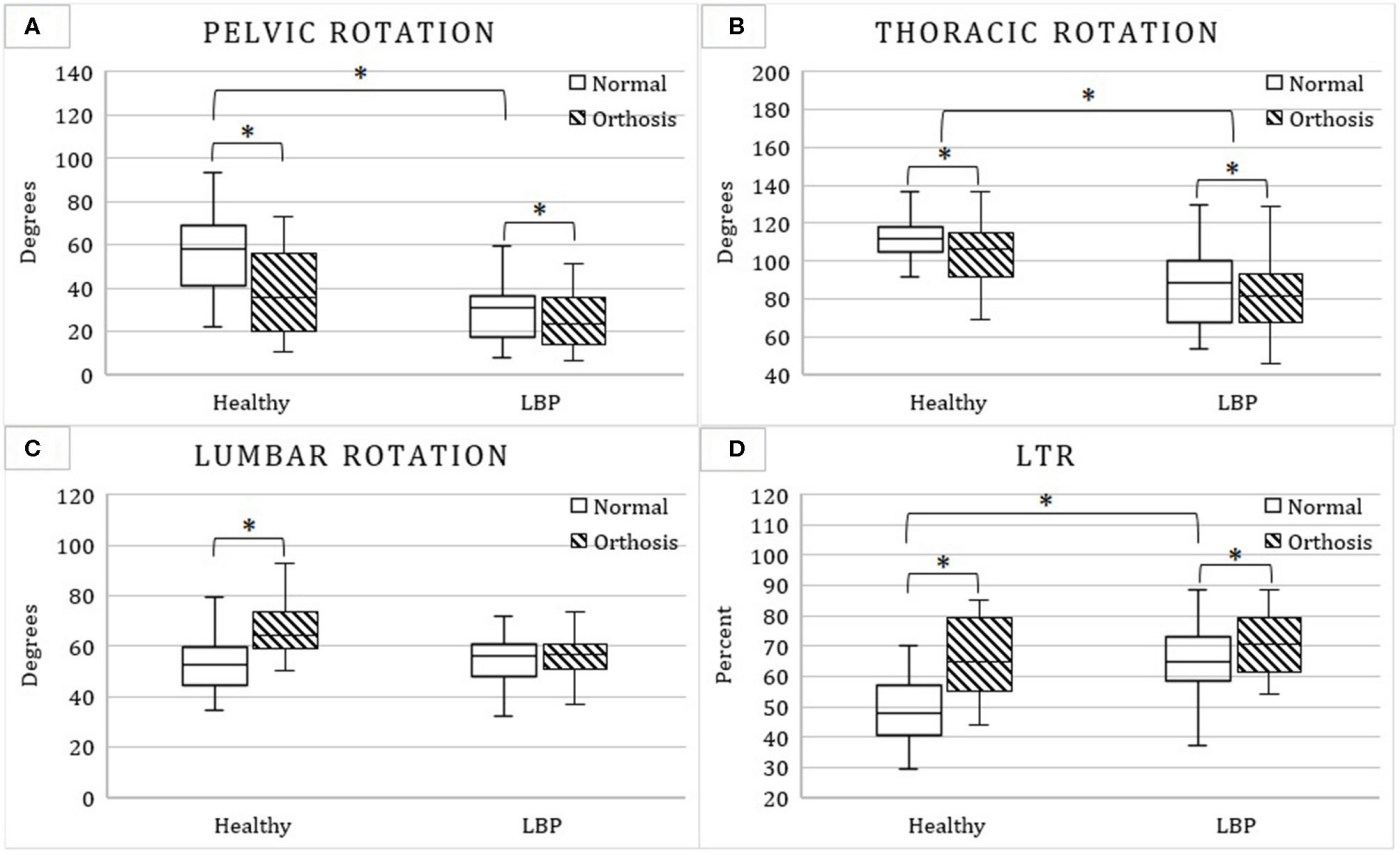
Mean and standard deviation of pelvic **(A)**, thoracic **(B)**, lumbar **(C)** rotations, and lumbo-thoracic ratio (LTR; **D**) with and without the hip orthosis among patients with low back pain (LBP) and asymptomatic controls. Stars indicate significant difference between means.

**Figure 3:**
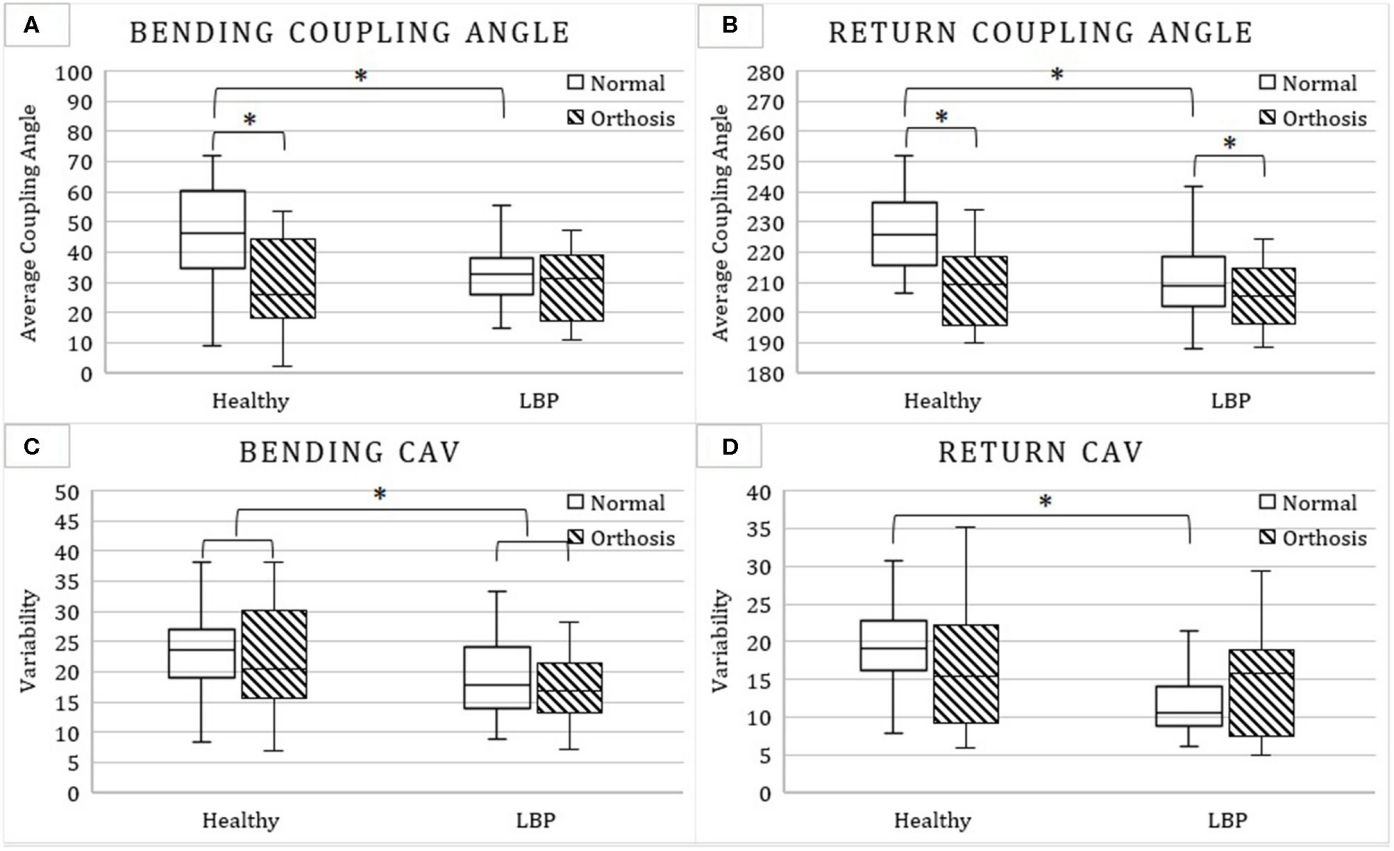
Mean and standard deviation of bending **(A)** and return coupling angle **(B)** as well as bending **(C)** and return coupling angle variability (CAV; **D**) with and without the hip orthosis among patients with low back pain (LBP) and asymptomatic controls. Stars indicate significant difference between means.

The results of the repeated measure, mixed factor ANOVA tests for measures representing the magnitude aspect, timing aspect, and coordination pattern of LPC are, respectively, summarized in Tables 2–**4**. The corresponding results for *post-hoc* analyses are further summarized in Figures 2,3.

**Table 2:** Mean values and summary of ANOVA tests for thoracic, pelvic, and lumbar rotations and lumbo-thoracic ratio (LTR).

	**Orthosis Condition**				**Group**				**Interaction**	
**Variable**	**Without**	**With**	***F***	***P*-value**	**Healthy**	**LBP**	***F***	***P*-value**	***F***	***P*-value**
Thoracic Rotation	98.18°	93.49°	25.261	**<0.001**	106.83°	84.84°	12.896	**0.001**	0.397	0.532
Pelvic Rotation	43.98°	31.29°	65.463	**<0.001**	46.68°	28.59°	11.876	**0.001**	16.52	**<0.001**
Lumbar Rotation	54.20°	62.20°	44.083	**<0.001**	60.15°	56.26°	1.219	0.276	23.08	**<0.001**
LTR	57.37%	68.42%	74.617	**<0.001**	57.73%	68.06%	7.872	**0.008**	16.22	**<0.001**

*P < 0.05 indicates significance. Bold values are used to highlight significant P-values*.

### Interactions Between Orthosis Condition and Group

#### Magnitude Aspect

Significant interactions between the orthosis condition and group factors were found for pelvic rotation, lumbar rotation, and LTR. Pelvic rotation was larger in the healthy group (56.2° [19.6°] vs. 31.7° [17.0°]), but only without orthosis. The orthosis caused an increase in lumbar rotation, but only for the healthy group (Table 2 and Figure 2C). In non-orthosis movement, LTR was smaller in the healthy group than the LBP group (49.6 [12.3] vs. 65.1 [11.8]; Table 2 and Figure 2D).

#### Timing Aspect

No significant interactions between the orthosis condition and group factors were found for MARP or DP during bending or return (Table 3).

**Table 3:** Mean values and summary of ANOVA tests for Mean Absolute Relative Phase (MARP) and Deviation Phase (DP) during forward bending and backward return.

	**Condition**				**Group**				**Interaction**	
**Variable**	**Without**	**With**	***F***	***P*-value**	**Healthy**	**LBP**	***F***	***P*-value**	***F***	***P*-value**
MARP Bending	19.47°	24.21°	2.705	0.108	20.91°	22.76°	0.297	0.589	2.631	0.334
MARP Return	19.45°	24.02°	2.699	0.109	24.66°	18.81°	2.059	0.16	9.862	0.113
DP Bending	12.45°	15.19°	2.727	0.107	13.78°	13.86°	0.002	0.964	0.609	0.44
DP Return	11.74°	14.07°	2.219	0.145	13.95°	11.86°	1.021	0.319	3.02	0.09

*P < 0.05 indicates significance*.

#### Coordination Pattern

Significant interactions between the condition and group factors were found for the bending and return coupling angles, and for return CAV. The orthosis caused a decrease of 17.9° in the bending coupling angle, only for the healthy group (Table 4 and Figure 3A). This shifted the coordination pattern from distal (pelvic) dominant to proximal (lumbar) dominant. Without the orthosis, bending and return coupling angles were larger in the healthy vs. LBP group (46.7° vs. 32.2°; Figure 3A and 227.3° vs. 210.7°; Figure 3B), indicating that the healthy group had a pelvic dominant movement pattern, and LBP group had a lumbar dominant movement pattern. Return CAV was larger in the healthy group (19.3° [5.7°] vs. 13.1° [7.0°]; Figure 3D), but only in non-orthosis condition.

**Table 4:** Mean values and summary of ANOVA tests for coupling angle and coupling angle variability (CAV) during forward bending and backward return.

	**Orthosis Condition**				**Group**				**Interaction**	
**Variable**	**Without**	**With**	**F**	***P*-value**	**Healthy**	**LBP**	***F***	***P*-value**	***F***	***P*-value**
Coup Angle Bending	52.06°	46.06°	26.098	**<0.001**	61.02°	37.10°	335.96	**<0.001**	15.35	**<0.001**
Coup Angle Return	219.02°	207.14°	405.47	**<0.001**	218.23°	207.93°	1451.4	**<0.001**	118.8	**<0.001**
CAV Bending	21.38°	20.93°	3.024	0.084	23.70°	18.62°	411.22	**<0.001**	5.397	0.365
CAV Return	16.23°	15.61°	17.969	**<0.001**	17.99°	13.86°	76.47	**<0.001**	202.7	**<0.001**

*P < 0.05 indicates significance. Bold values are used to highlight significant P-values*.

### Main Effects of Orthosis Condition on LPC

#### Magnitude Aspect

With orthosis pelvic rotation reduced from 44.0 to 31.3° (Table 2 and Figure 2A), thoracic rotation decreased from 98.2 to 93.5° (Table 2 and Figure 2B) and LTR increased from 57.4 to 68.4% (Table 2 and Figure 2D).

#### Timing Aspect

The orthosis was found to have no significant effects on the timing aspect of LPC (Table 3).

#### Coordination Pattern

Return coupling angle decreased with orthosis from 219 to 207° (Table 4 and Figure 3B). This indicates a shift toward a more lumbar dominant LPC pattern.

### Main Effects of Group on LPC

#### Magnitude Aspect

Compared to the healthy group, the LBP group had smaller thoracic rotation (106.8° vs. 84.8°; Table 2 and Figure 2B).

#### Timing Aspect

No significant difference was found between groups for the timing aspect of LPC (Table 3).

#### Coordination Pattern

The LBP group had a smaller bending CAV compared to the healthy group (23.7° vs. 18.6°; Table 4 and Figure 3C).

## Discussion

Abnormal LPC including larger pelvic and smaller lumbar rotations are widely reported for individuals suffering from LBP (Marras and Wongsam, 1986; Ahern et al., 1988; Paquet et al., 1994; Porter and Wilkinson, 1997; Shojaei et al., 2017a). This study sought a unique approach to LPC correction through use of a hip orthosis. The first objective was to determine if lumbar contributions (presented here as LTR) to trunk motion can be increased using a hip orthosis to restrict pelvic rotation. Our results supported the first hypothesis as orthosis was found to decrease pelvic rotation and result in an increase of LTR. Our second hypothesis, that the orthosis-induced changes in LPC would be more pronounced in individuals with current episodes or recent history of LBP, was not, however, supported by our results. On the exploratory aspect of the study and consistent with the orthosis-induced changes in magnitude aspect of LPC, the effects of orthosis on coordination pattern, wherever significant, was an increase in dominancy of lumbar contribution to trunk motion. Interestingly, however, timing aspect of LPC was not affected by application of the hip orthosis.

### Differences in LPC Between LBP and Healthy Individuals

The LBP group in this study exhibited smaller pelvic rotations, similar lumbar rotations, and higher LTR along with coupling angles that were more lumbar dominant when compared to the healthy group. These LPC abnormalities are different than the widely reported abnormalities in LPC that involve larger pelvic and smaller lumbar rotations along with pelvic dominant movement pattern when compared with back-healthy individuals (Marras and Wongsam, 1986; Ahern et al., 1988; Paquet et al., 1994; Porter and Wilkinson, 1997; Shojaei et al., 2017a). LBP patients have also reported to demonstrate smaller MARP and DP compared to healthy controls which has been suggested to indicate a self-guarding technique to reduce movement of the lumbar spine (Mokhtarinia et al., 2016), but no significant differences were found between the LPB and healthy group in this study. The only aspect of LPC of the LBP group found to be consistent with earlier literature were the CAV abnormalities. Specifically, wherever significantly different, CAV was smaller in patients with LBP compared to asymptomatic controls which is consistent with the reported less variability in a flexion-extension task among patients with LBP (Mokhtarinia et al., 2016). A possible reason for such contradictions between our findings and those reported in earlier study could be the participants' age. There is insufficient evidence concerning abnormalities in LPC of younger individuals suffering from LBP. This knowledge gap warrants further research as it has been suggested that LBP at a young age may have a role in development of chronic LBP in older ages (Negrini and Carabalona, 2002). The average age of patients with LBP evaluated in earlier research was much higher than those evaluated in this study (28–58 year means vs. 21). Comparing measurements of individuals with LBP with a mean age closer to ours (Porter and Wilkinson, 1997), the lumbar range of motion was similar to the mean lumbar rotation for all groups reported here (57.53° vs. 54.20°).

### Effects of Orthosis on LPC

Wearing the hip orthosis, as expected, reduced pelvic rotation and increased LTR. The orthosis-induced increase in LTR of patients with LBP was primarily due to the reduction of thoracic rotation with orthosis whereas in asymptomatic controls it was also influenced by an increase in lumbar rotation in addition to the reduction in thoracic rotation. Particularly, such an orthosis-induced increase in lumbar rotation of asymptomatic individuals increased their LTR to the extent that the difference in LTR between the groups observed in no-orthosis condition disappeared with orthosis (Figure 2D). The absence of orthosis-induced changes in lumbar rotation of patients with LBP could be due to the smaller rotation of pelvis observed among patients with LBP in this study that was affected less by the orthosis compared to the asymptomatic group (i.e., 6° vs. 19°). Therefore, using such a pelvic restraining mechanism in patients with LBP that demonstrate larger than normal pelvic rotation is likely to increase lumbar rotation as was originally expected and hypothesized in this study. The lack of change in lumbar rotation of LBP group with orthosis could also be due to fear of pain and an effort to avoid further aggravation of their LBP. The influence of fear-avoidance behavior of patients on orthosis-induced changes could, however, be understood better if the orthosis effects are investigated in a patient population with larger pelvic rotation than those evaluated in the present study.

The results on the coordination pattern also demonstrated some effectiveness of the orthosis in its intended use but again more so among asymptomatic individuals. Where significant, the orthosis caused a decrease in coupling angle, indicating a shift from pelvic dominant to lumbar dominant movement patterns. Similar to magnitude aspect, such observed impact on the coordination pattern could in part be due to the larger unconstrained pelvic rotation among asymptomatic individuals compared to LBP group.

The orthosis did not significantly change MARP, DP, and CAV during bending or return. While not statistically significant, average values for MARP and DP were, however, higher with vs. without the orthosis during both bending and return. Given that an increase in MARP and DP have been suggested to indicate a less guarded LPC (Mokhtarinia et al., 2016), the trends observed here give hope that future investigations into the use of a hip orthosis will not negatively impact the timing aspect of LPC.

Back orthoses (a.k.a. back belts) have been promoted as tools for treatment of LBP, particularly when the LBP is a result of an overuse injury. Similar to the hip orthosis investigated in this study, back belts can alter LPC but primarily by restricting the lumbar motion. Back orthoses have been reported to be capable of reducing lumbar motion between 24 and 64% (Cholewicki et al., 2003; Jegede et al., 2011; Larivière et al., 2014). The hip orthosis in our study was similarly found capable of decreasing pelvic rotation, a decrease that ranged between 19% (among patients) and 34% (among asymptomatic individuals) and appeared to depend on the magnitude of unrestricted pelvic rotation. Back orthoses were not found to affect pelvic rotation, but decreased thoracic rotation by 6 to 13° (Larivière et al., 2014) and 42 to 47% (Cholewicki et al., 2003).

### Implications

Investigations into the biomechanical effects of abnormal LPC show that it could be detrimental on the lower back (Tafazzol et al., 2014; Shojaei et al., 2018). Physical therapy techniques, including lumbar stabilization programs are popular treatment methods for LBP (Searle et al., 2015); however, Shahvarpour et al. found a lumbar stabilization program had no significant effects on LPC and that patients retained a lower lumbar spine range of motion (compared to healthy controls) after pain and disability had decreased (Shahvarpour et al., 2017). Functional restoration programs have shown some effectiveness in correcting LPC, with LBP patients capable of achieving a normal LPC reporting less pain than patients who don't (Mayer et al., 2009). These treatments do not work for all patients which leaves the need for alternative methods of LPC correction. While the orthosis used in this study has shown an ability to acutely improve LPC, there is still a need to determine if these effects could be persistent and play a role in reducing LBP recurrence particularly among patients whose abnormal LPC involves larger pelvic rotation.

### Limitations

A rather young patient population (18–28 years) was investigated in this study which limit the generalizability of our findings. Older patients have been reported to display LPC abnormalities contrary to those observed in this study. Therefore, orthosis-induced changes in LPC are likely to be different in older individuals suffering from LBP that have been reported to have larger pelvic and smaller lumbar rotations. Age-related differences in lower back biomechanics, particularly a larger resistance to passive deformation of the lumbar spine (Shojaei et al., 2016) and smaller lumbar contributions (Vazirian et al., 2017a), would likely influence orthosis-induced changes in LPC of older individuals. The differences in body mass between the two groups, while not significant, may suggest a different level of motion artifact in the IMU measurements due to soft tissue deformation. The potential effects of participant clothing on the orthosis' ability to restrict motion were not controlled. Participants were not provided with nor instructed to wear specific types of clothing. As such, the orthosis may have been applied tighter to some participants than others, which could have altered the amount of pelvic restriction. Finally, our results only reflected immediate effect of restraining pelvic rotation, the long-term impact of wearing such a hip-orthosis on LPC and LBP experience remain to be investigated in future.

## Conclusion

Given the high recurrence rate of LBP and considering that current LBP treatments have limited effectiveness in correcting LPC, the possibility remains that abnormal LPC could be a contributing factor to LBP. A novel approach was implemented to correct LPC through use of a hip orthosis. The hip orthosis increased relative lumbar contributions to a trunk forward bending and backward return task by physically restricting pelvic rotation. Contrary to our expectation, orthosis-induced changes in LPC were smaller in patients with LBP most likely because the LBP group evaluated in this study had smaller unrestricted pelvic rotations. Otherwise, larger orthosis-induced changes in LPC were observed in the group with larger pelvic rotations (i.e., asymptomatic individuals). Therefore, the effects of hip orthosis on older patients with LBP as well as the long-term impact of a hip orthosis on LPC and LBP experience worth future investigation.

## Data Availability Statement

The raw data supporting the conclusions of this article will be made available by the authors, without undue reservation.

## Ethics Statement

The studies involving human participants were reviewed and approved by University of Kentucky Institutional Review Board. The patients/participants provided their written informed consent to participate in this study.

## Author Contributions

MB and BB conceived the presented idea. MB and CD performed the experiments and analyzed the data. MB wrote the manuscript with critical feedback and support from CD and BB. BB supervised the project. All authors contributed to the article and approved the submitted version.

## Conflict of Interest

The authors declare that the research was conducted in the absence of any commercial or financial relationships that could be construed as a potential conflict of interest.
